# Relationship Between Prostaglandin and Interleukin Concentrations in Seminal Fluid and Their Influence on the Rate of Fertilization in Men Undergoing ICSI

**DOI:** 10.3390/ijms26157627

**Published:** 2025-08-06

**Authors:** Houda Amor, Fatina W. Dahadhah, Peter Michael Jankowski, Rami Al Nasser, Lisa Jung, Ingolf Juhasz-Böss, Erich Franz Solomayer, Mohamad Eid Hammadeh

**Affiliations:** 1 Biochemistry & Molecular Biology of Reproductive Medicine Laboratory, Department of Obstetrics, Gynecology & Reproductive Medicine, Medical Faculty, University of Saarland, 66421 Homburg, Germany; peter.jankowski@uks.eu (P.M.J.); erich.solomayer@uks.eu (E.F.S.); mehammadeh@yahoo.de (M.E.H.); 2 Department of Endocrinology & Reproductive Medicine, University Clinics Freiburg, 79106 Freiburg, Germany; lisa.jung@uniklinik-freiburg.de (L.J.); ingolf.juhasz-boess@uniklinik-freiburg.de (I.J.-B.); 3 Department of Basic Dental Sciences, Faculty of Dentistry, The Hashemite University, Zarqa 13115, Jordan; fwdhadha@hu.edu.jo; 4 British Syrian IVF Centre, Al-Rasheed Hospital, Damascus 0100, Syria; raminasser03@yahoo.com

**Keywords:** cytokines, fertilization, interleukins, male infertility, prostaglandin

## Abstract

Sperm count, motility, and morphology are semen parameters that directly affect male fertility. The presence of cytokines in seminal plasma negatively or positively influences these parameters. Interleukins and prostaglandins are proinflammatory cytokines present in human seminal plasma and play crucial roles in fertilization, in general and after intracytoplasmic sperm injection (ICSI) procedures. This study aimed to investigate the possible influence of interleukins IL-17 and IL-18, and prostaglandins PGE2 and PGF2α on male infertility. Semen samples were collected from 58 males who underwent the ICSI procedure. An enzyme-linked immunosorbent assay (ELISA) was used to determine the levels of IL-17, IL-18, PGE2, and PGF2α, and these concentrations were then correlated with semen parameters and the rate of fertilization. Furthermore, the chromatin integrity of the sperm was evaluated with an Acridine Orange (AO) assay. The results showed an inversely proportional relationship between the AO binding intensity and fertilization rate (r = −0.394; *p* ≤ 0.002). Furthermore, a negative correlation was observed between the IL-18 concentration and positive AO (*p* ≤ 0.021). Moreover, the IL-18 concentration was positively correlated with the fertilization rate (*p* ≤ 0.05). In contrast, IL-17 did not significantly correlate with any semen parameters or with the fertilization rate. Seminal PGE2 levels were significantly correlated with embryo cleavage at 72 h (*p* ≤ 0.05). To conclude, this study revealed that denaturation of sperm nuclear deoxyribonucleic acid (DNA) contributes to low fertilization rates. In addition, this study proposed a potential role for IL-18 in fertilization. PGE2 likely influences embryo development, but further studies are needed to examine the impact of seminal PGE2 on the oocyte to fully elucidate its contribution to this complex biological process.

## 1. Introduction

Infertility is a worldwide concern that affects approximately 15% of couples trying to conceive and ultimately seeking medical assistance for their fertility challenges. Male-related disorders contribute to 50% of these childless couples [1]. Male infertility is a significant factor in human reproductive failure [2]. Various causes of male infertility have been identified, including genetic mutations, erectile dysfunction, inflammatory diseases, chemotherapy, ejaculatory duct obstruction, varicocele, and sperm deoxyribonucleic acid (DNA) damage [3,4].

Semen quality is both an indicator and a predictor of male fertility. The World Health Organization (WHO) has established criteria for semen analysis, which are based on standard and classical parameters [5]. These parameters evaluate the functionality of the testes and sperm. However, it remains uncertain which specific parameter best reflects the integrity of the male reproductive process.

With advancements in genetic biotechnology, there has been a growing focus on studying DNA and chromatin in spermatozoa. Chromatin in human spermatozoa exists in a complex form. During spermiogenesis, sperm chromatin undergoes several modifications, including the replacement of histones with protamines [6]. This nucleo-protamine complex facilitates the formation of highly condensed genomic DNA [7]. The integrity of sperm chromatin is essential for proper sperm function as numerous studies have associated abnormalities in sperm chromatin with poor outcomes in assisted reproductive technology (ART) [8,9]. As a result, evaluating sperm chromatin integrity has been suggested as a practical approach for assessing the fertilization potential of sperm [10].

Males with fertility issues often display various nuclear abnormalities, including atypical chromatin structures, in their sperm [11]. Furthermore, DNA fragmentation is more prevalent in these individuals [12]. Elevated levels of sperm DNA damage have been linked to an increased risk of miscarriage after in vitro fertilization (*IVF*) and intracytoplasmic sperm injection (ICSI) procedures [13]. However, some studies indicate that even infertile males with significant DNA fragmentation can successfully achieve pregnancy through *IVF* and an ICSI [14,15].

Over the past few decades, ART has undergone numerous enhancements designed to improve fertilization, blastulation, and implantation rates. Several studies have highlighted the significance of seminal plasma in the context of ART. For example, in ICSIs, the use of mature sperm of the highest quality is essential. Seminal plasma is the primary component of semen and acts as a protective medium by providing sperm with the supplements needed for metabolism and proper function [16].

In addition, seminal plasma includes various immunoregulatory factors, primarily cytokines, immunoglobulins, and chemokines [17,18]. These components trigger immune responses in the female body after coitus, triggering an inflammation-like reaction [19]. Additionally, many studies indicate that seminal plasma greatly enhances implantation rates, probably owing to its varied cytokine composition, which boosts innate immune responses in women, including the recruitment of leukocytes [20,21].

Inflammation plays a major role in male infertility. Growing evidence proves that inflammatory cytokines and lipid mediators, more specifically prostaglandins (PGs), play a major regulatory function in male reproductive activity [22]. Cytokines are a large group of proteins primarily released by immune-competent cells and other cells in response to stimuli such as tissue infections [23]. They play a critical role in modulating immune cell activation, which includes processes such as proliferation, growth, differentiation, and mobility. In addition, cytokines are involved in various biological processes, facilitating signal transmission between immune cells [24,25]. In the male genital tract, cytokines are produced primarily by testicular macrophages, with Leydig and Sertoli cells also serving as significant sources of these proinflammatory mediators. Notably, these cells have been demonstrated to secrete most of interleukin 1 (IL-1) and interleukin 6 (IL-6) in the male gonad [26]. Cytokines are naturally present in human seminal fluid [27], and substantial evidence indicates that they play a vital role in regulating the development and normal functioning of the testes [28]. Cytokines typically do not operate in isolation but function within a complex network. Various studies have identified significant correlations among different cytokines [25,29,30]. It has been demonstrated that activated leukocytes migrate from the site of infection to the seminal plasma during inflammatory conditions, resulting in an increased presence of these cells in semen. It is likely that a higher concentration of leukocytes in semen affects semen parameters and influences the potential for successful pregnancy [31,32].

Interleukin-17 and Interleukin-18 belong to those cytokines recently receiving a fair amount of attention in the etiopathogenesis of male infertility. Homeostasis of the testicular immune privilege and normal spermatogenesis path is maintained by IL-17, a proinflammatory cytokine majorly produced by T helper cells known as Th17 cells [33]. Increased expression of this cytokine has recently been associated with immotile as well as nonviable sperm, which should logically have a negative impact on male reproductive potential [34].

IL-18 belongs to the cytokine family IL-1. It has also been involved in discussions on inflammation-related infertility [35]. Studies carried out were able to establish that IL-18 levels are associated with poor sperm parameters in urogenital-infected males [36,37] and negatively associated with pregnancy outcomes via IVF treatments [38]. Other major immunological functions that IL-18 performs include apoptosis and the induction of cytokines. These further place more theoretical relevance on this particular cytokine when discussing reproductive dysfunction. Notwithstanding these above findings, such a direct relationship between IL-18 levels and semen quality has not been adequately addressed in the earlier literature.

Prostaglandins (PGs) are active lipids that are produced from arachidonic acid (AA) through the action of cyclooxygenase (COX) enzymes and are classified as COX metabolites of AA [39]. They serve autocrine and paracrine functions that modulate gene transcription and intracellular signaling. Prostaglandin activity is triggered by binding to specific prostanoid G protein-coupled receptors. The enzyme phospholipase A2 releases AA from plasma membrane phospholipids, initiating the synthesis of prostaglandins [40,41]. Seminal fluid contains 15 distinct prostaglandins, with prostaglandin E being the most predominant. The majority of these prostaglandins are produced in seminal vesicles [42]. The four primary seminal prostaglandins include PGE1, PGE2, 19-hydroxy PGE1, and 19-hydroxy PGE2; additional components observed in seminal fluid include PGF1α, PGF2α, and PGE3 [43]. In particular, PGE2 has been demonstrated to promote the acrosome reaction in sperm as they are near the egg, aiding the entry of extracellular calcium into the cytoplasm of human sperm [44]. On the other hand, PGF2α also appears to promote motility in short-term incubations; for long-term incubations or at high concentrations, it seems to have deleterious effects. It acts differentially depending on its concentration [45,46]. This underscores the fact that optimal sperm function, and hence successful reproduction, requires a prostaglandin equilibrium. This is particularly highlighted when considering assisted reproductive techniques such as intracytoplasmic sperm injections (ICSIs).

Inflammation of the male genital tract is often associated with leukocytospermia and the production of reactive oxygen species (ROS), leading to oxidative stress and damage to sperm DNA wherein the fertilization rate and pregnancy outcome are reduced [47,48,49,50]. IL-17 and IL-18 together with prostaglandins as PGE2 and PGF2α do not only indicate the inflammatory status but can also be real mediators of these deleterious processes.

Though the presence of these mediators in seminal fluid has already been confirmed, very few studies have tried to establish direct relationships between them and the outcomes of fertilization, more so in ICSIs. This study, therefore, attempts to fill that gap by evaluating the seminal levels of IL-17, IL-18, PGE2, and PGF2α against major semen parameters, sperm chromatin integrity, and fertilization success rate among a group of men who are recipients of ICSIs. In doing so, it will go a long way toward unraveling the actual contributions of these proinflammatory factors to male fertility besides providing preliminary evidence on which factor may be considered a marker or therapeutic target toward improved reproductive outcomes.

## 2. Results

Semen parameters were analyzed in a total of 58 samples. Table 1 presents the means and standard deviations (STDs) for all examined semen parameters across these patients. The semen volume was consistently greater than 2 mL for each ejaculate. The average sperm concentration was 37.2 ± 37.4 m/mL. The mean percentage of motile sperm was 44.1 ± 23.6%, while the percentage of positively stained sperm via AO staining was 20.6 ± 16.8%. Additionally, the mean percentage of sperm exhibiting typical morphology was 2.4 ± 2.09%.

A statistically significant positive correlation was found between age and sperm count (r = 0.304; *p* ≤ 0.020). Additionally, semen volume was significantly correlated with normal sperm morphology (r = 0.276; *p* ≤ 0.036). The sperm count was positively correlated with sperm motility across the entire patient population (r = 0.304; *p* ≤ 0.002). Furthermore, a positive and statistically significant association was observed between sperm morphology and sperm count (r = 0.419; *p* ≤ 0.001). A notable correlation was also established between sperm morphology and sperm motility (r = 0.379; *p* ≤ 0.003).

**Figure 1:**
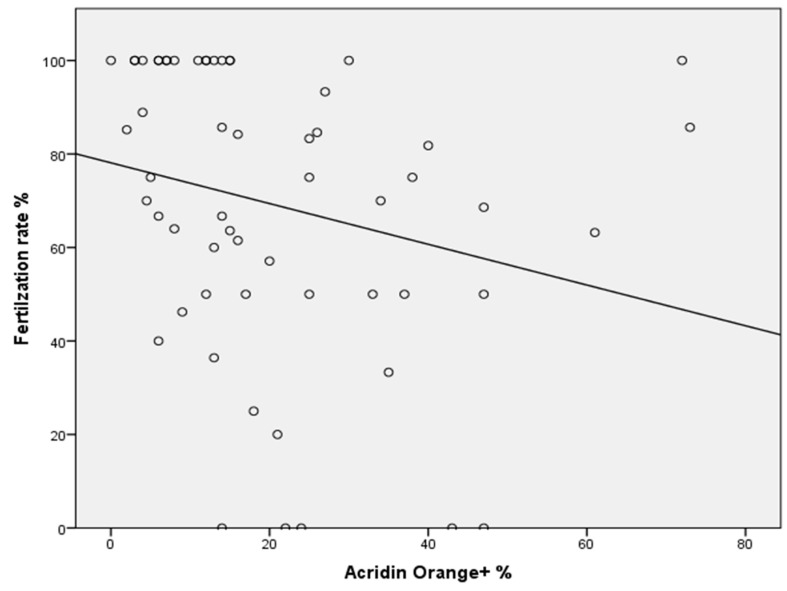
Negative correlation between the AO^+^ concentration and fertilization rate (*p* ≤ 0.002).

High-quality embryos typically divide reliably into 2 to 4 cells after 48 h and 6 to 8 cells after 72 h. We assessed the cleavage rate by dividing the number of embryos by the total number of fertilized eggs at each specific time point. The fertilization rate did not correlate with the sperm parameters, except for a negative association with positive AO (r = −0.394; *p* ≤ 0.002) (Figure 1). The levels of IL-17, IL-18, PGE2, and PGF2α were analyzed in a total of 58 seminal plasma samples. The mean levels of these four markers are shown in Table 2 and were measured in pg/mL

**Figure 2:**
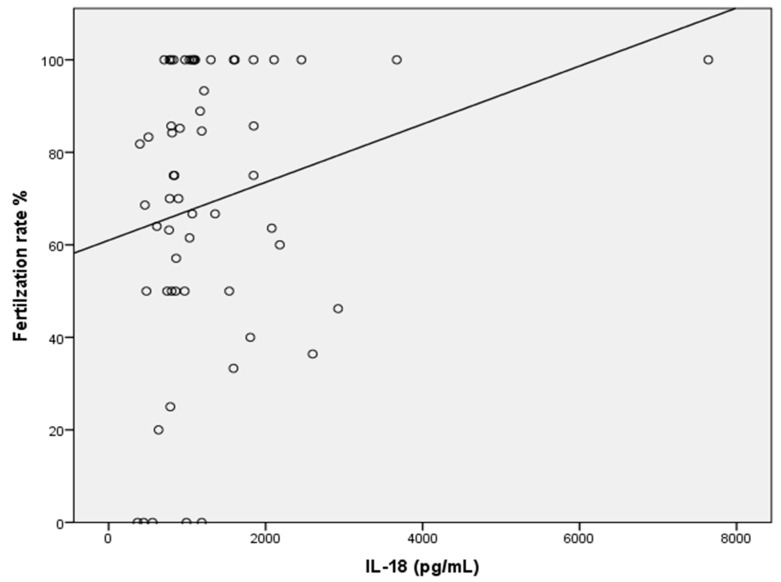
Positive correlation between the IL-18 concentration and fertilization rate (*p* ≤ 0.042).

The seminal IL-18 level was significantly correlated with the fertilization rate (r = 0.268; *p* ≤ 0.042) (Figure 2). Moreover, a negative association was observed between IL-18 and positive AO (r = −0.302; *p* ≤ 0.021). In contrast, IL-17 did not significantly correlate with the sperm parameters.

**Figure 3:**
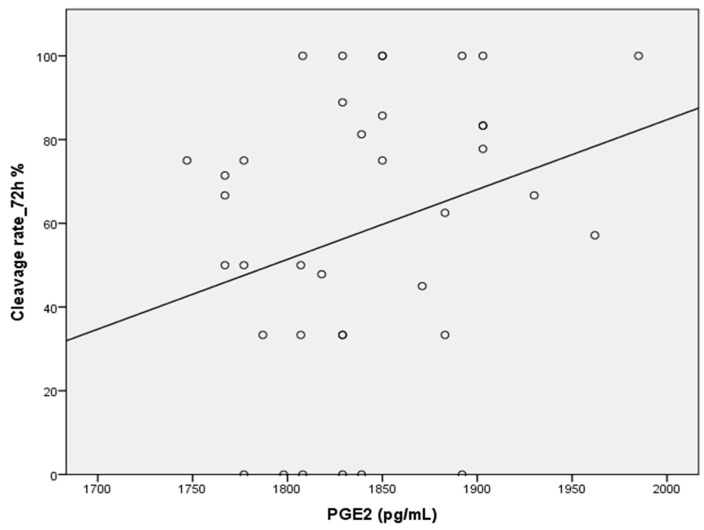
Positive correlation between PGE2 concentration and cleavage rate at 72 h (*p* ≤ 0.045).

**Figure 4:**
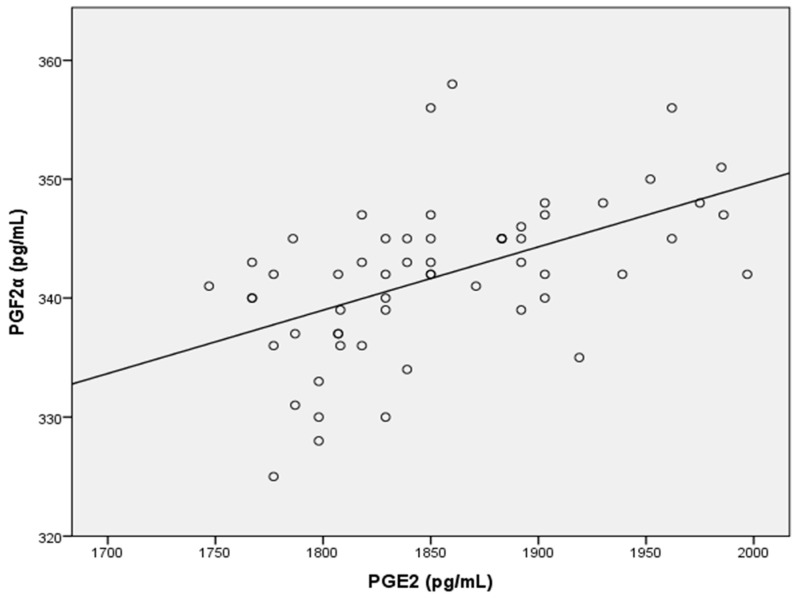
Positive correlation between PGE2 and PGF2α concentrations (*p* ≤ 0.0001).

Finally, PGE2 levels were significantly correlated with the cleavage rate at 72 h and with PGF2α (r = 0.332; *p* ≤ 0.045, and r = 0.580; *p* ≤ 0.0001, respectively) (Figure 3 and Figure 4). Conversely, no correlation was detected between PGF2α and any semen parameters.

## 3. Discussion

### 3.1. Impact of the Sperm Chromatin Structure on the Rate of Fertilization

Low ICSI success rates and failures in fertilization procedures are associated with impaired chromatin structures in ejaculates [51]. Numerous studies have revealed a significant relationship between sperm DNA fragmentation and low fertilization rates [52,53]. The use of sperm chromatin integrity as a diagnostic and prognostic indicator of fertilization rates before *IVF* and ICSIs remains a topic of debate [54]. However, previous findings have shown that the integrity of chromatin is a reliable predictive factor for the success of *IVF*, and it should be incorporated into sperm assessments along with conventional parameters for individuals undergoing *IVF* [55]. Furthermore, another study emphasized that chromatin structure abnormalities contribute to low fertilization rates [56].

In our study, we identified a negative correlation between AO positivity and fertilization rates, where positive AO indicates the presence of single-stranded DNA. Consequently, elevated levels of sperm DNA fragmentation may be directly linked to failure of fertilization. This observation aligns with numerous studies examining sperm chromatin compositions, suggesting that males with elevated amounts of single-stranded DNA experience reduced fertilization success [57,58].

### 3.2. Impact of Interleukins in Seminal Plasma on the Rate of Fertilization

In human cervical epithelial cells, exposure to semen triggers an inflammatory response in the female reproductive tract [19]. Seminal interleukins and prostaglandins significantly impact female tissues by inducing cellular alterations in the endometrium that promote embryo implantation and development. This process resembles a classical inflammatory cascade, during which the activation of immunoregulatory factors enhances endometrial receptivity and supports embryo implantation [59]. Furthermore, it has been shown that inflammatory cytokines play a vital role in sperm motility and their ability to achieve fertilization [60].

It has been demonstrated that seminal plasma cytokine profiles may be utilized to guide therapeutic strategies in reproductive medicine, ultimately improving pregnancy rates and outcomes in ART [61]. However, multiple studies have revealed that the levels of interleukins in the seminal plasma of infertile males are significantly greater than those in the seminal plasma of fertile controls [62,63,64]. This study explored the relationships between proinflammatory factors (specifically IL-17 and IL-18) and standard semen parameters, as well as fertilization rates.

The concentrations of IL-17 and IL-18 were measured in all semen samples, revealing no correlations between the levels of IL-17 and the classical semen parameters assessed in this study. However, a previous study reported elevated levels of seminal IL-17 in varicocele patients [61]. Additionally, another study demonstrated that low-quality sperm were associated with increased levels of IL-17 [65].

Interestingly, a negative correlation was found between the IL-18 concentration and AO positivity (*p* ≤ 0.021), suggesting that IL-18 may impact sperm chromatin integrity. In contrast, findings from an earlier study indicated higher IL-18 levels in fragmented chromatin structures in the semen of males experiencing infertility [37].

Further data revealed a significant positive correlation between the IL-18 concentration and the leukocyte count (*p* ≤ 0.006). This finding was expected, as macrophages mainly produce IL-18. Several studies have documented an association between high levels of leukocytes in semen and male infertility. Although leukocytes are normally present in human semen, they are considered pathological when their count exceeds 1 m/mL semen (leukocytospermia) [66]. This finding aligns with Matalliotakis’s conclusions, which illustrated that men with higher seminal leukocyte counts had increased IL-18 levels, suggesting that IL-18 levels could serve as a diagnostic marker for male genital tract infections [36]. In addition, we observed a positive correlation between the IL-18 concentration and the fertilization rate (*p* ≤ 0.042). The reason for this finding is not yet clear, but this observation prompted further investigation of seminal IL-18 and its potential contribution to successful pregnancies following ICSIs.

### 3.3. Impact of Prostaglandins in Seminal Plasma on the Rate of Fertilization

Prostaglandins (PGs) play various roles in the female reproductive system, with both PGE2 and PGF2α being essential for successful fertilization. Moreover, lower levels of prostaglandins in the human endometrium may result in decreased endometrial receptivity [67,68]. Most studies focused on prostaglandins have examined the effects of PGE2 and PGF2α on the endometrium during parturition. Elevated PGE2 levels have been observed in infertile men with low sperm counts and significantly impaired sperm motility [69]. Conversely, another study reported no PGE2-related effects on semen parameters [70]. Furthermore, the current findings also indicate no correlation between seminal PGE2 levels and traditional semen parameters.

No clear impact on the fertilization process in the early stages of the ICSI cycle has been identified concerning male prostaglandin levels. This study revealed a significant correlation between the PGE2 concentration and the cleavage rate at 72 h, suggesting that PGE2 plays an important role in embryonic development. These results align with those of a recent study showing that PGE2 is essential for successful fertilization [71].

Among all the biomarkers examined in this study, only PGE2 and PGF2α exhibited strong correlations, as evidenced by *p* values of less than 0.0001. It has been suggested that PGE2 directly influences effector T leukocytes to increase IL-17 production [72]. However, our study revealed no such correlation between IL-17 and PGE2. Further research is necessary to fully elucidate the roles of interleukins (IL-17 and IL-18) and prostaglandins (PGE2 and PGF2α) in fertilization and embryonic development. In addition, future studies with larger cohorts and receiver operating characteristic (ROC) curve analyses should aim to define such diagnostic thresholds for potential clinical application.

## 4. Materials and Methods

### 4.1. Samples Collection and Preparation

Semen samples (*n* = 58) from patients aged 22–52 years who underwent assisted reproductive therapy at the British Syrian IVF & Fetal Medicine Center of Al-Rashid Hospital in Damascus, Syria, were utilized in this study. The participants included men undergoing ICSI cycles, regardless of their fertility status. Informed consent from all participants was obtained and documented at the British Syrian IVF & Fetal Medicine Center prior to the initiation of any study-related procedures (BC-15933-22).

Patients with acute genital tract infections and autoimmune diseases were excluded based on their medical history. The patients provided ejaculate samples via masturbation, following an abstinence period of two to seven days. These samples were collected in polypropylene containers and allowed to liquefy at 37 °C for 20–30 min. After liquefaction, the ejaculate was assessed according to the WHO guidelines and prepared for use in intracytoplasmic sperm injections (ICSIs). Following centrifugation at 2500× *g* for 10 min, the upper layer of the ejaculate (seminal fluid without sperm) was stored at −20 °C for subsequent measurement of IL-17, IL-18, PGE2, and PGF2α, which did not exceed the three-month storage period. Standard semen analysis was conducted on all samples within one hour of collection according to the WHO criteria [1]. From each semen sample, two smears were prepared for morphology assessment based on Kruger’s strict criteria and analysis of chromatin integrity via Acridine Orange staining.

### 4.2. Acridine Orange (AO) Assay

**Figure 5:**
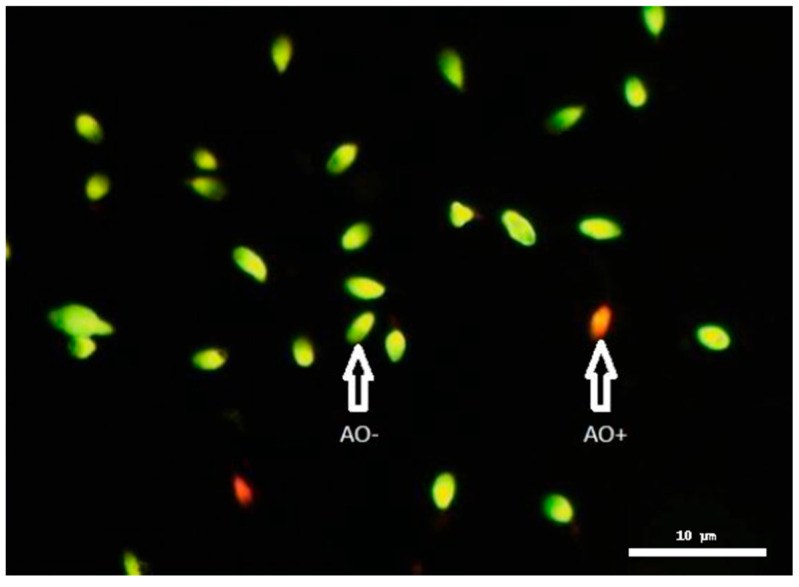
Human sperm stained with Acridine Orange (AO) at 100× magnification, distinguishing AO^−^ (green fluorescence: intact DNA, double-stranded DNA) from AO^+^ (red-orange fluorescence: denatured DNA, single-stranded DNA) sperm cells.

An Acridine Orange (AO) staining assay was performed to assess chromatin integrity in human spermatozoa, serving as an indicator of DNA fragmentation. Semen smears were fixed for 2 h in freshly prepared Carnoy’s solution (methanol/glacial acetic acid, 3:1), followed by air drying. The fixed slides were then stained with acid AO solution and evaluated under a fluorescence microscope (Figure 5). A total of 200 spermatozoa per sample were analyzed, distinguishing cells emitting orange fluorescence (denatured, single-stranded DNA-AO positive) from those emitting green fluorescence (intact, double-stranded DNA-AO negative), following the method of Virant-Klun [57].

### 4.3. Interleukin ELISA (IL-17 and IL-18)

Human IL-17 and IL-18 were detected and their quantity measured in seminal plasma using Human ELISA Kits, which can be obtained from R&D Systems (Minneapolis, MN, USA). The kit works based on the quantitative sandwich enzyme-linked immunosorbent assay (ELISA) principle. Standards and samples are added to wells already coated with a monoclonal antibody specific to interleukin, after which incubation takes place followed by washing. An enzyme-linked polyclonal antibody specific to the same interleukin is then added into each well. After a second period of incubation and another washing step to remove unbound conjugate, substrate solution is added for color development proportional to the bound interleukin amount. Absorbance was read at 450 nm after stopping the reaction with acid solution.

All samples were run in technical replicates, and standard curves were run using a 4-PL curve. The intra-assay CV was less than 10% and the inter-assay CV was less than 12%, thereby proving high precision of the assay as well as its reproducibility paralleling what has been suggested by the R&D Systems ELISA Guide. The range for IL-17 was from 15.6 to 2000 pg/mL, with a MDD lower than 15 pg/mL, and for IL-18 it was from 26.6 to 1700 pg/mL, with a MDD ranging between 0.539 and 7.52 pg/mL. IL-18 samples were prediluted fivefold before running them; hence, the final concentrations were measured values multiplied by the dilution factor.

### 4.4. Prostaglandin ELISA (PGE2 and PGF2α)

Prostaglandin PGE2 and PGF2α concentrations in seminal plasma were determined using competitive ELISA kits from Cayman Chemical (Michigan, MI, USA) according to the manufacturer’s instructions. The principle of this assay is based on the competition between free prostaglandins present in the sample and a limited number of prostaglandin-acetylcholinesterase (PG-AChE) conjugate for a defined number of binding sites of a specific PG monoclonal antibody. The antibody–PG complex will bind to wells coated with polyclonal IgG. After incubation, the plate was washed to remove any unbound reagent, and Ellman’s Reagent was added. A yellow color developed, which could be read at 412 nm and was inversely proportional to the amount of free prostaglandins present in the sample.

All samples and standards were run in duplicate. The standard curve was generated by plotting %B/B_0_ values against prostaglandin concentrations with a 4-parameter logistic (4-PL) fit. Typically, the intra-assay CV is <10% and the inter-assay CV is <15%. This occurs within specs provided by the manufacturer. The plates for PGE2 were incubated at room temperature for 1 h while PGF2α plates were incubated at 4 °C for 18 h.

### 4.5. Statistical Analysis

Statistical analysis was conducted via SPSS software (version 14.0) (SPSS Inc., Chicago, IL, USA). The correlation coefficient was used to analyze the relationships between the interleukin and prostaglandin levels and semen parameters. A *p* value of ≤ 0.05 (two-sided) was considered statistically significant.

## 5. Conclusions

Seminal plasma contains various proinflammatory and immunomodulatory factors that may be beneficial for fertilization, implantation, and pregnancy. However, an imbalance of cytokines in seminal plasma could result in infertility and miscarriage.

To the best of our knowledge, this is among the very few studies that attempt to relate these four inflammatory mediators and their combined effect on major semen parameters, sperm chromatin integrity (AO staining), and fertilization outcome under ICSI conditions. Other studies before this have been carried out in isolation and outside the context of assisted reproduction.

A negative correlation between IL-18 and AO-positive sperm is observed. A positive correlation appears between IL-18 and the fertilization rate. It lets one think of the potential protective or modulatory effects of IL-18. Clinical studies do not document this very well.

We noted a very marked relationship between the levels of PGE2 in seminal plasma and embryo cleavage at 72 h post-fertilization. Prostaglandins, more particularly PGE2 and PGF2α, are known to elicit motility as well as transport responses on sperm; however, their association with the results of early embryonic development following ICSIs has not been adequately explored.

It suggests that IL-18 and PGE2 levels in seminal plasma could be evaluated as possible predictive markers of ART success. This becomes a steppingstone toward the development of cost-effective, non-invasive diagnostic tools to make clinical decisions in the evaluation of male fertility.

To sum up, what makes this work new is not only the specific biomarkers that were studied but the combined assessment, direct linkage to ICSI outcomes, and potentiality as predictive tools in the context of assisted reproduction.

## Figures and Tables

**Table 1:** Descriptive characteristics and statistical data for all men (*n* = 58).

Characteristics	Mean	Minimum	Maximum	STD
Age (year)	38.4	22.00	52.00	6
Volume (mL)	2.9	0.50	5.00	1.2
Count (m/mL)	37.2	0.00	150	37.4
Motility (% motile)	44.1	0.00	80.00	23.6
Leukocytes	0.3	0.00	4.00	0.9
Morphologically normal spermatozoa (%)	2.4	0.00	8.30	2
Acridine Orange (positive AO%)	20.6	0.00	73.00	16.8
Oocyte fertilization rate (%)	69.1	0.00	100.00	30.9
Cleavage rate 48 h (%)	86.45	0.00	100.00	29.7
Cleavage rate 72 h (%)	58.36	0.00	100.00	33.8

**Table 2:** The average concentrations of IL-17, IL-18, PGE2, and PGF2α in all patients.

Characteristics	Mean	Minimum	Maximum	STD
IL-17 (pg/mL)	10.8	3.6	153	19.4
IL-18 (pg/mL)	1299	368	7640	1073
PGE2 (pg/mL)	1854	1747	1997	63.7
PGF2α (pg/mL)	341	325	358	6.5

## Data Availability

The data presented in this study are available on request from the corresponding author. The data are not publicly available due to ethical restrictions.
